# Supplementary material for: Mapping transgene insertion sites reveals the α-Cre transgene expression in both developing retina and olfactory neurons
Source: Commun Biol. 2022 May 3;5:411. doi: 10.1038/s42003-022-03379-9 (PMC9065156; doi:10.1038/s42003-022-03379-9)
Supplement: Supplementary file 2 — Supplementary Information [file 42003_2022_3379_MOESM2_ESM.pdf]

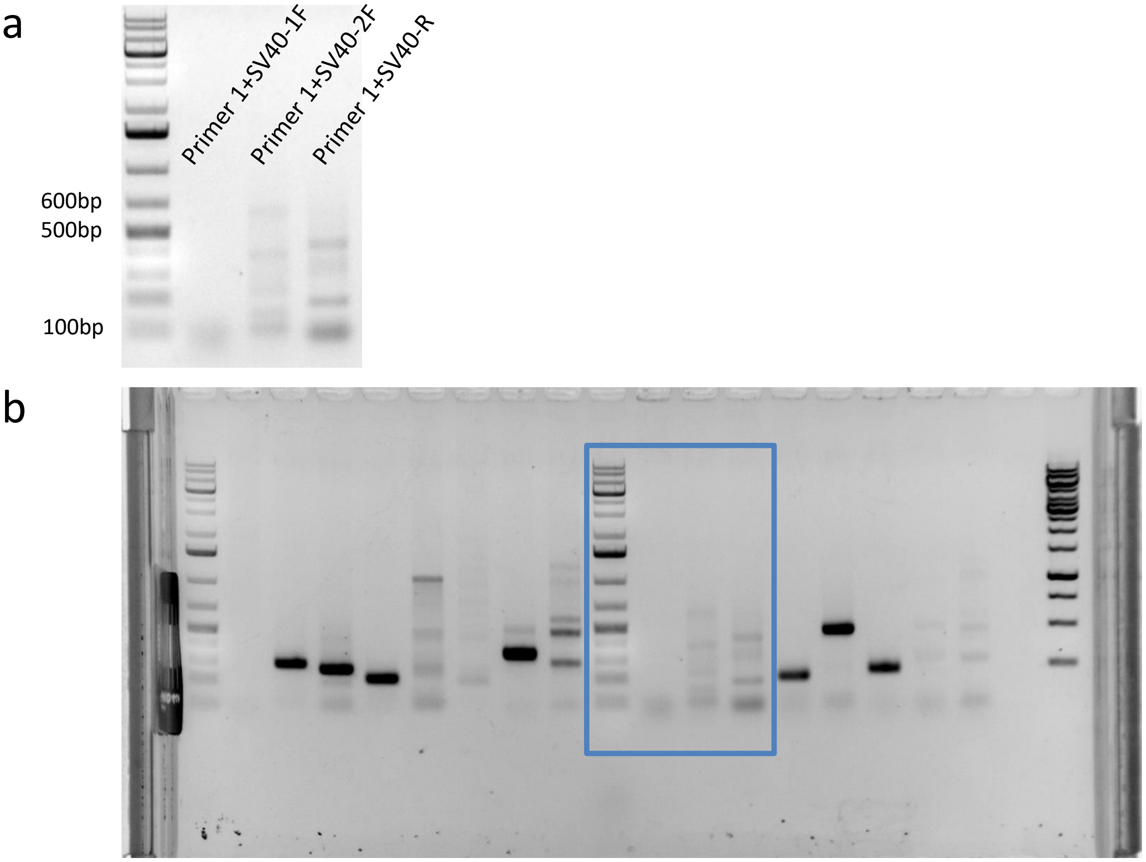

Supplementary Figure 1. The PCR results using primer 1 and some primers in the SV40 polyA region.

(a) Primer 1 targets the left junction but combining it with several primers from the SV40 polyA region (Supplementary Table 3) did not generate any specific PCR products.

(b) Uncropped gel images for (a).

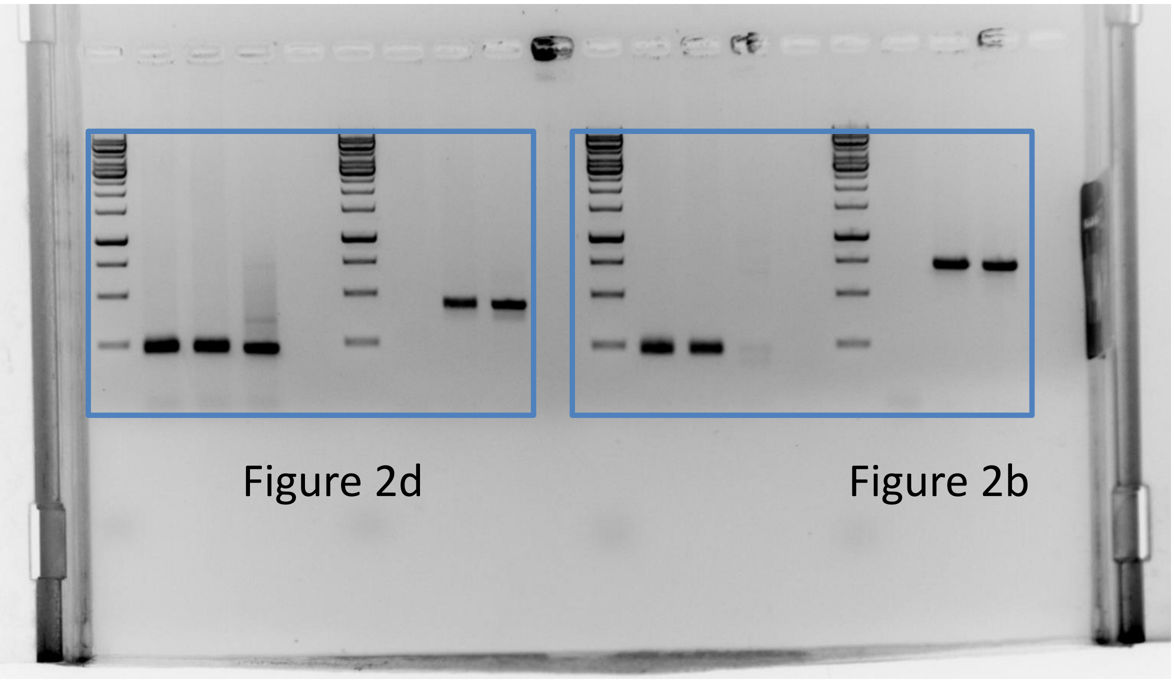

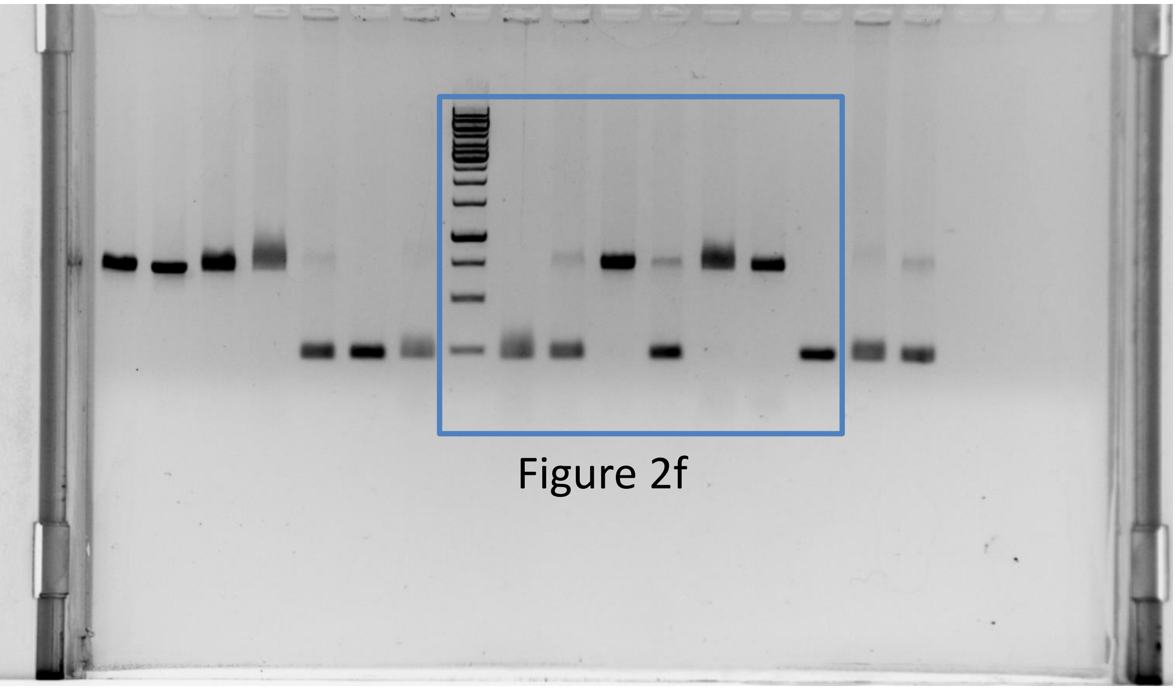

Supplementary Figure 2. Uncropped gel images for Fig 2b, 2d, 2f.

Figure 2. Confirmation of the transgene insertion site on Chromosome 7, and genotyping of zygosity.

(b) Primers 1 and 2 were used to confirm the 27 kb deletion. The 250 bp band is absent in putative homozygous α-Cre animals. Primers 1 and 5 were used to confirm the left junction between the 3′ end of the α-Cre transgene and the 5′ end of Chromosome 7 in homozygous animals. This 722 bp band is absent in wild-type animals.

(d) Primers 3 and 4 were designed to confirm the right-hand junction. However, the 250 bp band is still present in putative homozygous α-Cre animals (arrow). Primers 4 and 6 were used to confirm the right junction between the 5′ end of the α-Cre transgene and the 3′ end of Chromosome 7 in homozygous animals. This 436 bp band is absent in wild-type animals.

(f) Genotyping of zygosity of the α-Cre transgene using primers 1+2+5 from the left junction. Totally 7 animals were genotyped, including 2 wild types, 2 hemizygotes and 3 homozygotes.

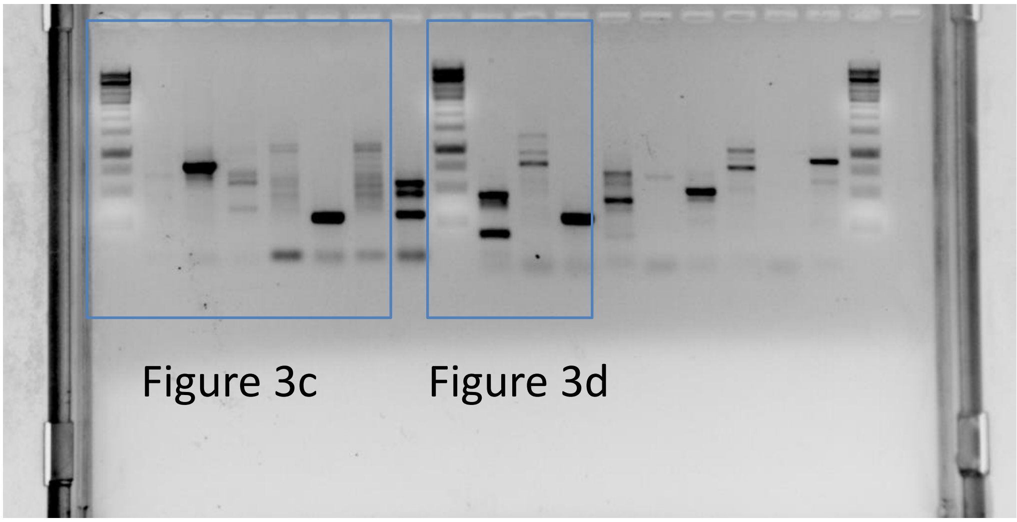

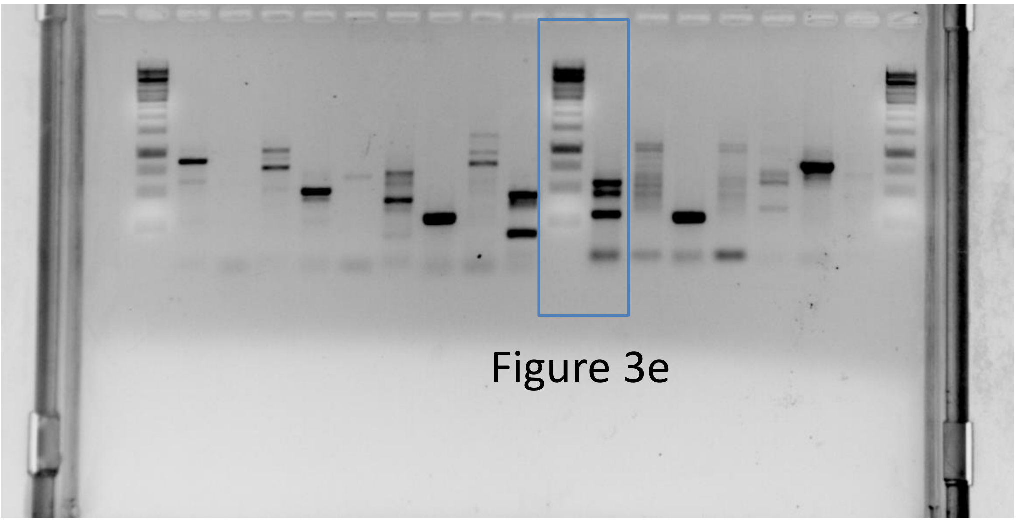

Supplementary Figure 3. Uncropped gel images for Fig 3c-e
Figure 3. Evidence for four tandem transgene copies.
(c) Primers from the *Hbb* intron (F66, F36) and SV40 polyA signal (SV40-1F, -2F, -R) confirmed that the SV40 signal is downstream of the *Hbb* intron.
(d) Primers from the α-enhancer (R351) and SV40 polyA signal (SV40-1F, -2F, -R) confirmed the existence of tandem transgene copies. Blue arrows indicate multiple bands with R351/SV40-1F, but only one band with R351/SV40-2F primers.
(e) Primers from *Hbb* intron (F36) and the α-enhancer (R351) confirmed three versions of the SV40/α-enhancer junction.

Supplementary Table 1 Primers for TLA analysis

Primer Set	Name	Sequences
Primer set 1	RV461	5´-ATTACGTATATCCTGGCAGC-3´
Primer set 1	FW907	5´-GGAGTTTCAATACCGGAGAT-3´
Primer set 2	RV330	5´-AACCAGTGAAACAGCATTG-3´
Primer set 2	FW408	5´-GTTCGAACGCACTGATTTC-3´

Supplementary Table 2 Primers for differentiating zygosity of α-Cre transgene

Primer	Location	Junction (Figure 2a)	Sequence
Primer 1	Chromosome 7	Left junction	TGAATCCTGCCTATCATGTG
Primer 2	Chromosome 7	Left junction	CCCCAAAATTCCTCAAAAGA
Primer 3	Chromosome 7	Right Junction	ATTGAGATGGGCTGCTCTGT
Primer 4	Chromosome 7	Right Junction	GCTCCATCTTCACCCCTGTA
Primer 5	*α-Cre* transgene	Left junction	CACCATGGACCCCATGATAA
Primer 6	*α-Cre* transgene	Right Junction	AATTGCATCGTCACGACAAA

Supplementary Table 3 Primers to map the entire transgene sequence

Amplified for	No.	Primer	Primer sequence（5’→3’）
Breakpoint at α-enhancer	1	F40	GATGCCCATCAGGAGACAGT
	2	F154	GCTCCATCTTCACCCCTGTA
	3	WT R382	ATTGAGATGGGCTGCTCTGT
	4	WT R471	AAACGTTTTGAAGGGGGAGT
	5	R308	AGGGCAAGGCCTACAGAAAG
	6	R351	GCAAGAGTGGACATTGCACA
	7	R379	ATCTGGGCTCTGGCTGTCT
	8	R473	AATTGCATCGTCACGACAAA
	9	R841	TTTCCACTTCCTCGGACATC
	10	R1164	TTGGAAATCCCACTCACACA
Breakpoint at *Hbb* intron	1	R540	TGAATCCTGCCTATCATGTG
	2	WT F5	GGGAAATCAGGGATACAGAGG
	3	WT F257	CCCCAAAATTCCTCAAAAGA
	4	U1R	TCTCTTTCCTACAGCTCCTTGG
	5	F66	GGGCAAAGTTTTCAGGGTGT
	6	F115	CACCATGGACCCCATGATAA
	7	F36	GTGCTGGTTGTTGTGCTGTC
	8	F175	TCAGCCTTCCAGAACTGTTG
SV40 polyA	1	SV40-1F	ACCTGAAACATAAAATGAATGCAA
	2	SV40-2F	ACCGTCGACCTCGAGAAGTA
	3	SV40-R	CCAGACATGATAAGATACATTGATGA
Linker-1	1	BRAZ11-F	GGATATTAAGGAAAGTTAGCGCC
	2	BRAZ20-F	TCGTTGACATTTAAACTCTGGGGCAGGTCCTCGCG
	3	BRAZ12-R	CCGGTTATTCAACTTGCACCATGCCGCCC
	4	BRAZ19-R	GAACCTCATCACTCGTTGCATCGACCGG
Linker-2	1	IRES-R134	GGGAAAGACCCTAGGAATGC
	2	IRES-R188	GAGGAACTGCTTCCTTCACG
	3	Cre-F228	GCGCTAAGGATGACTCTGGT
	4	Cre-F323	ATACCGGAGATCATGCAAGC
Linker-3	1	GFP-R160	ATGGCGCTCTTGAAGAAGTC
	2	GFP-R278	TTCCCTTAAGCTCGATCCTG
	3	IRES-F393	AATGGCTCTCCTCAAGCGTA
	4	IRES-F401	TAACAAGGGGCTGAAGGATG
Linker-4	1	GFP-F518	CCCAACGAAAAGAGAGACCA
	2	GFP-F285	TCAAGGAGGACGGAAACATC
	3	BG-R071	AATCAAGGGTCCCCAAACTC
	4	BG-R122	CCATGGTGATACAAGGGACA

Supplementary Table 4 Primers for genotyping

Mouse	Primer	Sequence	WT product	Transgene product
α-Cre	Cre-F	ATGTCCAATTTACTGACCG		724bp
	Cre-R724	CCCGGCAAAACAGGTAGTTA		
R26R	R1295	GCGAAGAGTTTGTCCTCAACC	550bp	250bp
	R523	GGAGCGGGAGAAATGGATATG		
	R26F2	AAAGTCGCTCTGAGTTGTTAT		
Ai14	oIMR9020	AAGGGAGCTGCAGTGGAGTA	297bp	200bp
	oIMR9021	CCGAAAATCTGTGGGAAGTC		
	oIMR9103	GGCATTAAAGCAGCGTATCC		
	oIMR9105	CTGTTCCTGTACGGCATGG		

Supplementary Table 5 Primers for RT-PCR of olfactory genes on Chromosome 7

Genes	Forward primers	Reverse primers
vmn2r67	accatagcaccaaaggcaag	tatagcatttgggggcaaaa
vmn2r68	gccacatcatcattgtttgc	cctggccaagaaagctacag
vmn2r69	tggatacatgggctctgttg	aagagcttggcctcattgaa
vmn2r70	gccacatcatcattgtttgc	cctggccaagaaagctacag
Olfr 291	tgcgctatccactctttgtg	gtcacaatggccaagaggat
Olfr 310	ccttgtcattgcaagtgtgc	tcagcaaagcaggaagatca
OMP	ctgcagttcgatcactggaa	cgtgtcatgaggttggtgag
