# Supplementary material for: Mapping transgene insertion sites reveals the α-Cre transgene expression in both developing retina and olfactory neurons
Source: Commun Biol. 2022 May 3;5:411. doi: 10.1038/s42003-022-03379-9 (PMC9065156; doi:10.1038/s42003-022-03379-9)
Supplement: Supplementary file 3 — Description of Additional Supplementary Files [file 42003_2022_3379_MOESM3_ESM.pdf]

# Description of Additional Supplementary Files

**File name:** Supplementary Data 1
**Description:** Source data behind Figure 3g, 590 Figure 7a-c.
