# Supplementary material for: Mapping transgene insertion sites reveals the α-Cre transgene expression in both developing retina and olfactory neurons
Source: Commun Biol. 2022 May 3;5:411. doi: 10.1038/s42003-022-03379-9 (PMC9065156; doi:10.1038/s42003-022-03379-9)
Supplement: Supplementary file 5 — Reporting Summary [file 42003_2022_3379_MOESM5_ESM.pdf]

nature portfolio

Corresponding author(s): Rod Bremner, Danian Chen

Last updated by author(s): Mar 25, 2022

Reporting Summary

Nature Portfolio wishes to improve the reproducibility of the work that we publish. This form provides structure for consistency and transparency in reporting. For further information on Nature Portfolio policies, see our Editorial Policies and the Editorial Policy Checklist.

Statistics

For all statistical analyses, confirm that the following items are present in the figure legend, table legend, main text, or Methods section.

n/a	Confirmed	
☐	☒	The exact sample size (*n*) for each experimental group/condition, given as a discrete number and unit of measurement
☐	☒	A statement on whether measurements were taken from distinct samples or whether the same sample was measured repeatedly
☐	☒	The statistical test(s) used AND whether they are one- or two-sided<br>*Only common tests should be described solely by name; describe more complex techniques in the Methods section.*
☐	☒	A description of all covariates tested
☒	☐	A description of any assumptions or corrections, such as tests of normality and adjustment for multiple comparisons
☐	☒	A full description of the statistical parameters including central tendency (e.g. means) or other basic estimates (e.g. regression coefficient) AND variation (e.g. standard deviation) or associated estimates of uncertainty (e.g. confidence intervals)
☐	☒	For null hypothesis testing, the test statistic (e.g. *F*, *t*, *r*) with confidence intervals, effect sizes, degrees of freedom and *P* value noted<br>*Give P values as exact values whenever suitable.*
☒	☐	For Bayesian analysis, information on the choice of priors and Markov chain Monte Carlo settings
☒	☐	For hierarchical and complex designs, identification of the appropriate level for tests and full reporting of outcomes
☒	☐	Estimates of effect sizes (e.g. Cohen's *d*, Pearson's *r*), indicating how they were calculated

Our web collection on statistics for biologists contains articles on many of the points above.

Software and code

Policy information about availability of computer code

Data collection	Labeled cells were visualized and images were captured using a Nikon C1si laser scanning confocal microscope or Zeiss Axio Imager Z2 fluorescence microscope. Measurements were performed with Nikon NIS-Elements AR 3.10 software.
Data analysis	GraphPad Prism version 9.0

For manuscripts utilizing custom algorithms or software that are central to the research but not yet described in published literature, software must be made available to editors and reviewers. We strongly encourage code deposition in a community repository (e.g. GitHub). See the Nature Portfolio guidelines for submitting code & software for further information.

Data

Policy information about availability of data

All manuscripts must include a data availability statement. This statement should provide the following information, where applicable:

- Accession codes, unique identifiers, or web links for publicly available datasets
- A description of any restrictions on data availability
- For clinical datasets or third party data, please ensure that the statement adheres to our policy

The α-Cre transgene sequence (from α-enhancer to Hbb intron) and flanking chromosome 7 sequence have deposited to GenBank, accession number: MZ890862. Uncropped gel images for Fig 2b, 2d, 2f, and 3c-e are provided as supplementary figures. The source data behind the Figure 3g, Figure 7a-c in the paper are in Supplementary data 1. All other data are available from the corresponding author) on reasonable request.

Field-specific reporting

Please select the one below that is the best fit for your research. If you are not sure, read the appropriate sections before making your selection.

☒ Life sciences ☐ Behavioural & social sciences ☐ Ecological, evolutionary & environmental sciences

For a reference copy of the document with all sections, see nature.com/documents/nr-reporting-summary-flat.pdf

Life sciences study design

All studies must disclose on these points even when the disclosure is negative.

Sample size	In our study, the sample size for the RT-PCR experiment (Figure 7A) was chosen based on common practice in mice experiments, different genotypes were compared between at least three litters. The sample sizes for the transgene copy number experiment (Figure 3g) and the buried food pellet tests (Figure 7b, c) were estimated by the formula $n= [2(Za+Z1-\beta)2] /SES2$ (Bagiella and Chang, 2019; Festing, 2018; Kadam and Bhalerao, 2010). Type I error (alpha) is set as 0.05, type II error (beta) is set as 10-20%, and it is a two-sided effect. The SES (or Cohen's d) is the ES (effect size) divided by the pooled SD (standard deviation), so it is the magnitude of the difference between the means of two groups in units of SDs (Festing, 2018).
Data exclusions	No data were excluded.
Replication	Findings were reliably reproduced across at least three litters
Randomization	The experimental approaches did not require samples to be randomized. Samples, and the subsequent data collection and analysis, were handled the same way in all experiments.
Blinding	The investigators were not blinded to group allocation during data collection or subsequent analysis. This approach is considered standard for biochemical and microscopy experiments done in this study.

Reporting for specific materials, systems and methods

We require information from authors about some types of materials, experimental systems and methods used in many studies. Here, indicate whether each material, system or method listed is relevant to your study. If you are not sure if a list item applies to your research, read the appropriate section before selecting a response.

Materials & experimental systems

n/a	Involved in the study	
☐	☒	Antibodies
☒	☐	Eukaryotic cell lines
☒	☐	Palaeontology and archaeology
☐	☒	Animals and other organisms
☒	☐	Human research participants
☒	☐	Clinical data
☒	☐	Dual use research of concern

Methods

n/a	Involved in the study	
☒	☐	ChIP-seq
☒	☐	Flow cytometry
☒	☐	MRI-based neuroimaging

Antibodies

Antibodies used	Ap2a (Santa Cruz, SC-8975), Cre (Cell signaling, 15036), GFP (Abcam, ab6673), Ga0 (Upstate, 07-634), Gai2 (Abcam, ab157204), Ki67 (BD science Pharmingen, 14-5698-82), mCherry (Novus Biological, NBP2-25158), OMP (Abcam, ab183947), Pax6 (Covance, PRB-278P). Donkey anti-rat Alexa-488 (ThermoFisher A21208), Donkey anti-rabbit Alexa-568 (ThermoFisher A10042), Donkey anti-goat Alexa-647(ThermoFisher A21447), Donkey anti-chicken Alexa-594 (Jackson ImmunoResearch 703-585-155)
Validation	Validations of the primary antibodies are provided on the manufacturers' website

Animals and other organisms

Policy information about studies involving animals; ARRIVE guidelines recommended for reporting animal research

Laboratory animals	Tg(Pax6-cre,GFP)2Pgr (α-Cre) mouse (MGI:3052661), R26R mice (the Jackson Laboratory, stock 003474), Ai14 mice (the Jackson Laboratory, stock 007914), Wild type ICR mice. Both male and female. From E12-adults.
Wild animals	This study did not involve with wild animals.
Field-collected samples	This study did not involve field-collected samples.

Ethics oversight	All animal procedures were reviewed and approved by the Animal Care Committee (ACC) of University Health Network (UHN), Toronto, Ontario, Canada (AUP#1411), and the Ethical Review Committee of Animal Research of West China Hospital, Sichuan University, Chengdu, Sichuan province, China (AUP# 2018008A)

Note that full information on the approval of the study protocol must also be provided in the manuscript.
